# Electrochemical and Structural Property of TiSiNb TFSOC on Affordable Interconnects in Proton Exchange Membrane Fuel Cell Applications

**DOI:** 10.3390/nano10102010

**Published:** 2020-10-12

**Authors:** Saman Khosravi H., Rudolf Vallant, Lukas Ladenstein, Klaus Reichmann

**Affiliations:** 1 Institute for Chemistry and Technology of Materials, Graz University of Technology, Stremayrgasse 9, 8010 Graz, Austria; lukas.ladenstein@tugraz.at; 2 Institute of Materials Science, Joining and Forming, Graz University of Technology, Kopernikusgasse 24/I, 8010 Graz, Austria; rudolf.vallant@tugraz.at

**Keywords:** PEM fuel cell, interconnect, thin-film, corrosion resistance, sol–gel method

## Abstract

High cost and low electrochemical stability of the interconnection in Proton Exchange Membrane Fuel Cell (PEMFC) in the presence of H_2_SO_4_ are one of the main issues hindering the commercialization of these devices. This manuscript presents the utilization of cost-effective steel in an attempt to minimize the PEMFC interconnection costs with a thin-film solid oxide coating (TFSOC) providing sufficient corrosion resistance for efficient long-term operation. Novel Ti_0.50-y/2_Si_0.50-y/2_Nb_y1,2_O_2_ as TFSOC was deposited on the C45E steel as a metal interconnect utilizing a sol–gel process at various annealing temperatures. The analysis of the phase and surface morphology demonstrates that lower annealing temperatures developed nanometric crystallite size of 68 nm, more uniform structure and higher corrosion resistance. Under standard test conditions, the TFSOC demonstrated high polarization resistance (1.3 kΩ cm^2^) even after 720 hours (h). Electrical conductivity of the TFSOC as low as 1.4 × 10^−2^ (Ω m)^−1^ and activation energy of 0.20 eV were achieved, which helps to maintain the PEMFC output power.

## 1. Introduction

**Figure 1:**
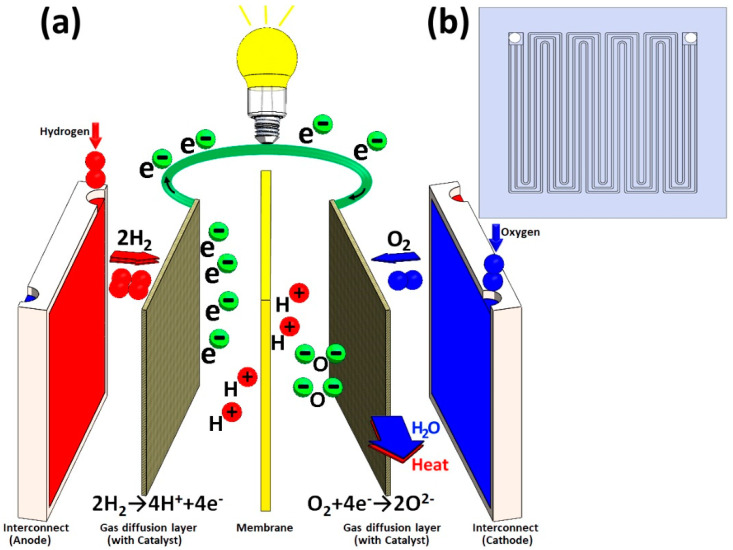
Schematic of Proton Exchange Membrane (PEM) fuel cell components (**a**) and front plane of Proton Exchange Membrane Fuel Cells (PEMFC_MI_) (**b**).

Proton Exchange Membrane Fuel Cells (PEMFC) are getting significant attention with the growing need for renewable energy and are expected to play a key role in the energy economy that is aiming for a green energy future [1]. A PEMFC is an electrochemical device that uses a hydrated Nafion membrane which is capable of converting hydrogen and oxygen to electrical energy. Schematic representation of a single stack cell of PEMFC with the highlighting of vital components and processes involved is shown in Figure 1a.

In this whole energy conversion process, water is the only produced by-product, making it of great interest to alternative energy research and to replace fossil fuels [2]. Having this in mind, the development and utilization of economical and reliable fuel cells are considered to be critical aspects of attaining genuinely green or sustainable energy [3]. PEMFC is regarded as a safe and most suitable cell of choice for industrial purposes because of its modest operation temperature. Currently available PEMFC can be operated between 80 °C and 200 °C [4]. The PEMFC interconnector (PEMFC_I_), also known as the flow field or separator is a vital component of PEMFC stack (depicted in Figure 1b) both in terms of weight and cost of the PEMFC encompassing about 80% of the total weight and 45% of the production cost of the stack [5]. These interconnectors are designed to perform several functions, such as gas flow channels, prevent hydrogen and oxygen from mixing in the fuel cell, water, and heat transmission, preventing leakage of reactants and coolant in addition to ultimately provide electrical connections [6]. The investigation of suitable and low-cost material for interconnect has emerged as a key factor in the development of the PEMFC stack. Additionally, it is important that the PEMFC_I_ is manufactured with lightweight materials using cost-effective techniques to keep the total cost of the cell stack low. At present, materials such as electro graphite [7], carbon–carbon composite [8], flexible graphite foil [9], polymer composite [10] and metallic sheets [11] are used for PEMFC_I_ applications. When it comes to commercial graphite PEMFC_I_, they show outstanding resistance to corrosion and favorable electrical and thermal conductivity. However, the low density, weak mechanical strength, material and processing cost restrict its usage [12,13,14]. Metal plate-based interconnectors are usually coated with various protective materials to enhance their corrosion resistance [15,16]. The common steel compositions used for PEMFC metal interconnects (PEMFC_MI_) are 304 and 316 stainless steel, which demonstrate reasonable resistance to corrosion but are costly [15]. On the other hand, these materials with good mechanical and thermal properties with a thickness of approximately 1–3 mm can significantly improve the performance and energy density of the cells [17] making the PEMFC_MI_ the best potential materials for PEMFC interconnects [18]. Nevertheless, one of the serious drawbacks of PEMFC_MI_ is its vulnerability to corrosion in a harsh PEMFC environment containing SO_4_^2−^, Cl^−^, F^−^, etc. [11] that limits the efficient long-term performance of PEMFC [19]. The corrosion of the PEMFC_MI_ leads to contamination of the membrane electrodes. It can be suppressed by passivation that in turn increases the interfacial contact resistance (ICR) and decreases the efficiency of the fuel cell stack. In recent studies, PEMFC_MI_ coated with carbon-based material has been reported as a cost-effective approach to improving corrosion resistance while keeping the ICR low [20,21]. Electrically conductive polymers (ECP) were studied as potential coating materials due to their chemical and electrochemical stability [22,23]. The presence of micro-defects, as a matter of the main disadvantage of ECP, can be used as migration channels for corrosive ions to penetrate to the substrate surface and passivate the interconnect, which could further increase the ICR [24,25]. Recently, some chemical inert conductive coatings, for instance, thin-film metallic glass coating (TFMGs) [26] and thin-film solid oxide coating (TFSOC) [27] have been considered as the most promising candidate coatings for PEMFC_MI_ applications. The TFMGs increase the price of PEMFC due to the high cost of equipment operating and limited area [28]. TFSOC has been primarily developed to resist tribo- corrosive degradation of metal surfaces in corrosive environments. Consequently, TFSOC can be a successful applicant to satisfy the clearly stated requirements to improve the corrosion resistance of the PEMFC_MI_ [29,30]. Therefore, developing cheap corrosion-resistant thin-film with appropriate electrical properties is crucial to keep the cost of PEMFC affordable. Nowadays, some TFSOC such as graphene/TiO_2_ [29] chitosan-tin oxide composite film [30], Ta_2_O_5_ [31], Nb-TiO_2_ [32], SnO_2_ [33], FTO [34], Cr_2_O_3_/C [35], PbO_2_ [36] and Zn-Ni-Al_2_O_3_ [37] have been investigated. These coatings have shown promising results in PEMFC_MI_ applications due to their high performance as efficient corrosion inhibitors. Nevertheless, further work needs to be done to improve TFSOC to achieve a reliable and affordable coating for application in PEMFC. So far there are some studies on the application of TiO_2_-SiO_2_ TFSOC for corrosion protection of different substrates [38,39]. TiNb-oxide TFSOC has shown reasonable conductivity and high chemical stability at room temperature (RT) [40,41]. To summarize, the TiO_2_, SiO_2_ and Nb_2_O_5_ protective coatings have become anti-corrosive and conductive modifier materials due to of their non-toxic characteristic, pollution-free, inexpensive and good chemical stability [38,39,40,41]. Sol–gel is proven to be a fairly simple and economical method for fabricating different organic and inorganic TFSOC for corrosion protection [42]. One main advantage of this method is its flexibility to coat large substrates of metal, glass, ceramics and plastic with a high purity protective layer using the dip-coating sol–gel technique [43]. The C45E carbon steel (AISI/SAE1045) is a commercial low-cost material (economic efficiency) commonly used due to its high strength and combination of formability that makes it easy to shape into sheets. However, several studies have been successfully performed to improve its electrochemical weakness such as corrosion resistance due to lack of chromium deficiency to operate under harsh conditions [44]. Our previous study has shown that TiO_2_-SiO_2_ TFSOC and its combination with C45E iron oxides after heat treatment process have successfully served as high physical and electrochemical protective barriers (61 kΩ cm^2^ compare to the 2 kΩ cm^2^ as polarization resistance for the bare C45E steel) against corrosive attacks [38]. Bai et al. [45] applied chromate coating on a C45E steel surface for application as a PEMFC_MI_ due to its excellent mechanical and cost-effective properties. The results demonstrated substantial protection at low and most stable corrosion current density with lowest interfacial contact resistance of 5.9 kΩ cm^2^, which indicated that the coated C45E has a significant potential to be used as PEMFC_MI_ applications. Therefore, it is important to assess the potential of TFSOC on C45E surface to combine the merits of TFSOC and substrate as a low-cost alternative interconnects in PEMFC. In this work, we focused on producing a cost-effective and environmental sustainable anti-corrosive TFSOC with long-term stability for use in PEMFC_MI_ applications. For this purpose, Ti_0.50-y/2_Si_0.50-y/2_Nb_y1,2_O_2_ (TiSiNb) as TFSOC were deposited via a sol–gel process on C45E steel substrates. The influence of Nb concentration and annealing temperature (500 °C, 600 °C and 700 °C) on the morphology of the thin films were examined. The microstructure and phase of obtained TFSOC were investigated. The electrochemical degradation behavior by means of overpotential application and electrical conductivity of TFSOC through impedance analysis were also investigated.

## 2. Experimental Procedure

### 2.1. Materials and Substrate Preparation

Tetra-n-butyl orthotitanate (TBOT, C_16_H_36_O_4_Ti, 98%, Merck, Billerica MA, USA) Tetraethyl orthosilicate (TEOS, C_8_H_20_O_4_Si 99%, Merck, Billerica, MA, USA) and Niobium (V) ethoxide (C_10_H_25_NbO_5_, 99.95%, Sigma-Aldrich, Taufkirchen, Germany) were used as precursors for titanium, silicone and niobium, respectively. As a solvent, hydrolysis agent and catalyst ethanol (C_2_H_5_OH, 99.99%, Merck, Billerica, MA, USA), distilled water and nitric acid (HNO_3_, 99.99%, Merck, Billerica, MA, USA) were used, respectively. The C45E steel with the chemical composition in accordance with EN 10083-2:2006 standard as illustrated in Table 1 was used as the reference sample (RS). Different silicon carbide abrasive papers (80 ground up to 4000 grit) and alumina with grain sizes of 1 μm and 3 μm (for surface polishing) were used to prepare the RS surface before applying TFSOC. Eventually, ethanol was used to clean the polished RS surface using an ultrasonic bath for 30 min.

### 2.2. Preparation of TiSiNb Sol

Two Ti_0.50-y/2_Si_0.50-y/2_Nb_y1,2_ (y = 0.01 and 0.02) sols with different Niobium (V) ethoxide (Nb) content (0.01 and 0.02) were prepared via the sol–gel method at RT. To synthesize coatings sols, TBOT, TEOS and Nb were used as precursors. Initially, to prepare the solution (A), 10 mL of TBOT was mixed with 20 mL absolute ethanol by utilizing a magnetic stirrer for 1 hour (h) at RT. The solution (B) comprising 5.8 mL of TEOS and 11.6 mL absolute ethanol was mixed in the beaker and stirred for 1 h at RT. In two separate beakers, two hydrolysis agents containing 5 mL distilled water and 2 mL nitric acid with 10 mL of absolute ethanol under severe agitation conditions (agent A) and 2.9 mL distilled water, 1.16 mL nitric acid and 5.8 mL of absolute ethanol (agent B) were stirred for 30 min at RT. Subsequently, the hydrolysis agents were added drop-wise under stirring to the A and B sols, respectively. In this step, 132 μL (y_1_ = 0.01) of Nb was added dropwise to the sol (A). Following the same procedure as before, the same sols and hydrolysis agents have been prepared for another doped Nb (y_2_ = 0.02) with 266 μL (pH-3 was measured). Eventually, the sols were maintained under intense stirring for 2 h to hydrolyze the precursors and aged for 24 h before deposited on the RS surfaces as TFSOC at RT.

### 2.3. Preparation of TFSOC on the Substrate

In order to deposit novel TiSiNb TFSOC, the layer-by-layer (LbL) technique [46] was used as an appropriate multi-layer coating production method due to its low cost, flexibility and durability. For this purpose, the cleaned RS was dipped at a constant rate of 10 cm/min in the TiSiNb aged sol. Following the full immersion after 2 min, the samples were pulled out from aged sol at the same constant rate speed and dried for 10 min at RT. To create the TiSiNb TFSOC three-layer (3L) this process was repeated three times. Eventually, after drying at 100 °C (3 °C/min) for 15 min the samples were annealed for 2 h at different annealing temperatures in a nitrogen atmosphere (N_2_) using a heating and cooling rate of 3 °C/min as outlined in Table 2.

### 2.4. Characterization of TFSOC

To identify the chemical decomposition of TiSiNb TFSOC precursors, thermal gravimetric analysis (TGA) and differential thermal analysis (DTA) were used. Approximately 10 mg of pre-basic material (dried gel at 100 °C for 12 h) was put within the TGA instrument (Model Netzsch, Selb, Germany) to determine the sample thermal stability and appropriate the annealing temperature. The experiment was conducted in the air atmosphere at RT up to 1000 °C (3 °C/min). In order to investigate the functional groups and molecule transplantation type of the chemical structure, a Alpha FT-IR spectrometer (Model ALPHA, Bruker, Ettlingen Germany) was acquired for the Fourier Transform Infrared Spectrum (FT-IR), which is equipped with a single-reflection ATR diamond module from ALPHA Platinum. Data were gathered between 400 to 4000 wavenumbers (cm^−1^) with a nominal resolution of 2 cm^−1^ in a total of 200 spectra. The composition of annealed gel (powder) and TFSOC were identified by using X-ray diffraction (XRD) (MiniFlex 600 Rigaku, Tokyo, Japan) using Cu Kα radiation operated at an accelerated potential of 40 kV and the current of 15 mA with the range of 2θ = 20–80° and step width of 0.01°. The outcomes were analyzed utilizing PANalytical XPert HighScore software to accurately determine the phases and measure the crystal-size obtaining in the full width at half maximum (FWHM). The surface morphology of TFSOC was investigated by using electron microscopy (TESCAN EDS + EBSD field emission) in the secondary electron mode (10 kV). Energy-dispersive X-ray spectroscopy (EDS) was utilized to obtain microanalysis of Rs and TFSOC. To investigate the RS and SOTFC corrosion behavior, potentiodynamic electrochemical polarization (PEP) with an initial potential scanning rate of −200 to 1000 mV (1 mV/s) versus open circuit potentials (OCP) and electrochemical impedance spectroscopy (EIS) tests were performed in standard condition solution (SCS) of 0.5 M H_2_SO_4_ with 2 ppm HF solution in 80 °C after 1, 24, 360 and 720 h. The over-saturated calomel electrode (SCE), platinum electrode and RS with and without TFSOC (surface area = 0.95 cm^2^) were employed as a reference electrode, auxiliary electrode and working electrode (WE), respectively. The EIS measurements were carried out at the stable OCP using AC signals (amplitude of 10 mV) and measurement frequencies from 10 mHz to 100 kHz. This technique focuses on the calculation of alternating current (ac) impedance across in the frequency range for corrosion velocity analysis. Therefore, small potential time variables were applied sinusoidally around the corrosion potential to measure the impedance (Z) of the system. The PEP and EIS of the RS and TFSOC were performed according to ASTM G3-14 [47] using Autolab model PGSTAT302N, Nova 1.11 and ZSimpWin 3.22 software to determine the current density (I_corr_), corrosion potential (E_corr_) in the range of ±25 mV, polarization resistance (R_p_) and TFSOC resistance (R_ct_). To analyze the conductivity and activation energy (Ea) of Ti_0.50-y/2_Si_0.50-y/2_Nb_y1,2_O_2_, the Ti-Si-Nb gel was dried, compacted to pellets of 13 mm diameter by uniaxial pressing and fired at 700 °C in N_2_ atmosphere. After that, gold pads were vapor-deposited on the surfaces of the pellets and impedance measurements at different temperatures between 100 °C and 700 °C were performed. A Novo control Concept 80 broadband dielectric spectrometer was used, which covered a frequency range from 0.1 Hz to 10 MHz. The conductivity was frequency independent up 1 MHz. The temperature was controlled by a thermocouple, which was positioned near the surface of the sample. For the determination of the conductivity at each temperature, the resulting Nyquist plots were fitted by a constant phase element (CPE) in parallel with a resistance element using the software ZView.

## 3. Results and Discussions

### 3.1. Thermal Gravimetric and Differential Thermal Analysis

**Figure 2:**
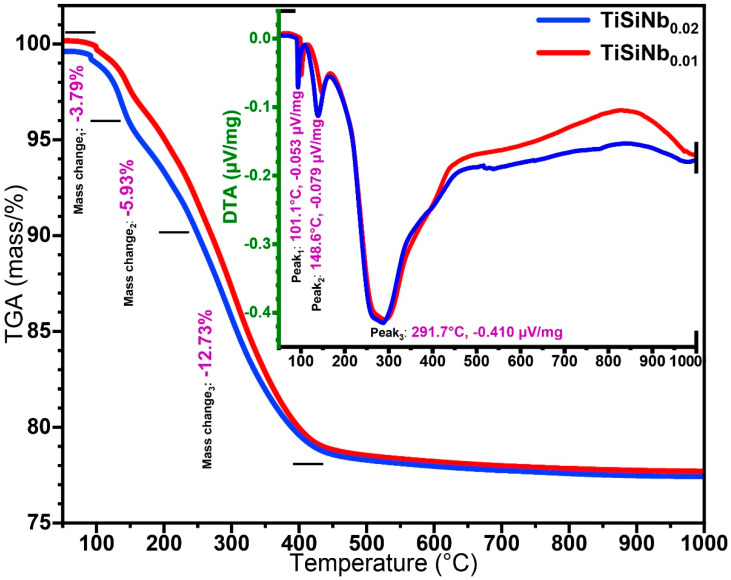
Thermal gravimetric analysis (TGA)-differential thermal analysis (DTA) curves of the TiSiNb dried gel at 100 °C for 12 h with different Nb contents.

Figure 2 demonstrates TGA-DTA curves of TiSiNb dried gel for 12 h at 80 °C containing different Nb content. Due to the low amount of niobium compared to other elements the results of 0.01 and 0.02 were not substantially different. The results indicate that the decomposition process of dried gel occurs in three steps. Over three steps, the overall weight loss percentage was approximately 23% of the primary precursor weight. Notwithstanding the aforementioned quantity, another weight loss occurred as a result of the removal of residual water from the TiSiNb gel in the drying process [48]. However, in the first step, approximately 3.79% of weight loss through evaporation of ethanol or combustion of organic compounds occurred between 75 °C to 175 °C [49]. The second step in 175 °C to 275 °C with a weight loss of 5.93% is ascribed to the removal of residual Ti–OH and Si–OH (unreacted hydroxyl groups) as well as carbonization [50]. The final step was a maximum weight reduction of around 12.73% at 400 °C up to 900 °C, which represents the transition of a crystalline titanium phase and physically releases water molecules bound to the silica-titanium interface region [51]. DTA data showed three significant changes (Exothermic peaks), which were contributed to the mass reduction in the dried gel. The oriented peaks at 76 °C and 150 °C were attributed to evaporation of physically adsorbed water [52] and approximately at 570 °C the greatest peak was caused by the phase transformation of titanium from amorphous to anatase [53]. Consequently, TGA-DTA dried gel analysis may infer that the TiSiNb TFSOC transmission phase (annealing temperature) ought to be between 500 °C and 600 °C.

### 3.2. FT-IR Spectra of TiSiNb TFSOC

**Figure 3:**
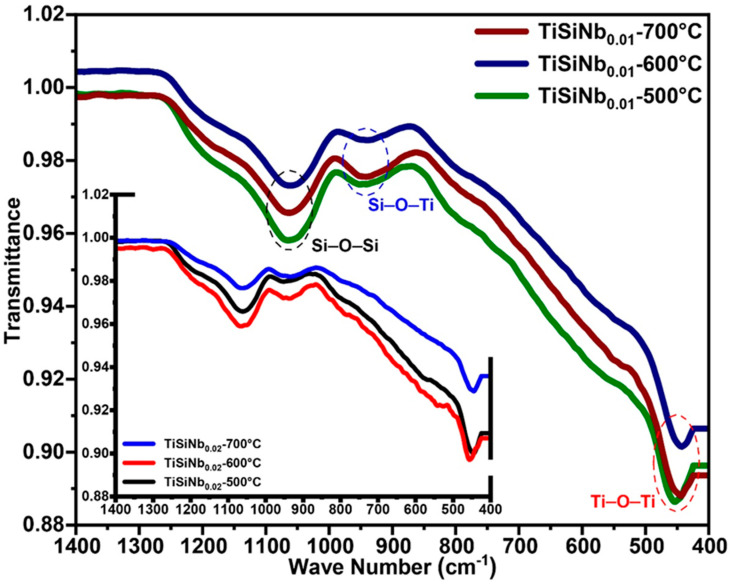
FT-IR spectra of TiSiNb annealed gel in N_2_ atmosphere at different temperatures and Nb contents.

The most recommended technique for evaluating Ti–Si bands is infrared absorption spectroscopy (FT-IR). The FT-IR spectra of TFSOC are shown in Figure 3.

No peaks were observed in the range of 1400 cm^−1^ to 4000 cm^−1^. It is commonly accepted that surface water adsorbed as well as the stretching vibration of OH and H_2_O bands are accessible in this range [39]. This indicates that a significant fraction of the aforementioned groups exists but they have not manifested because of the low intensity of peaks and annealing atmosphere, which allows water to gradually escape the pores. The vibrations of the Ti–O–Ti and Ti–O bands connection in titanium (anatase) were assigned between 463 cm^−1^ to 468 cm^−1^ and 517 cm^−1^, respectively [39,54]. Hydrolysis of the alcoholic group to produce Ti–OH occurs as a result of nucleophilic substitution of alkyl groups such as O–R. According to Equations (1)–(3) the –OH hydroxyl and condensation groups of Ti–OH might be produced the Ti–O–Ti with H_2_O and ROH, which contributed to the formation of the gel.

(1) $${\mathrm{Ti}\;(\mathrm{OR})}_{n}+{\mathrm{nH}}_{2}O\;\to \;{\mathrm{Ti}\;(\mathrm{OH})}_{n}+\mathrm{nROH}$$

(2) $${\mathrm{Ti}\;(\mathrm{OH})}_{n}\;\to \;{\mathrm{Ti}(O}_{n/2})+n/2\;H_{2}O$$

(3) $$\mathrm{TiOR}\;+\;\mathrm{TiOH}\;\to \;{\mathrm{TiO}}_{2}+\mathrm{ROH}$$

Furthermore, the peaks identified at 920 cm^−1^ and 950 cm^−1^ are correlated with the Si–O–Ti and Si–OH tensile vibration and the signal at 1050 cm^−1^ is referred to the Si–O–Si band, respectively [55,56]. The lack of Nb–O peak could be attributed to the low content of Niobium (V) ethoxide precursor in the sol. Therefore, all of the above results collected from the FT-IR analysis demonstrate that TiO_2_ and SiO_2_ have been copolymerized contributing to the formation of a homogenous inorganic hybrid xerogel.

### 3.3. SEM-EDS Analysis

**Figure 4:**
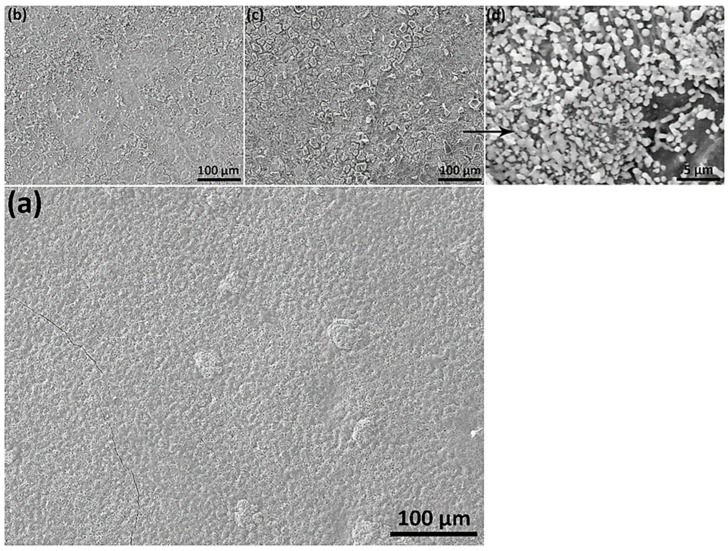
SEM micrographs of TiSiNb_0.02_ TFSOC: (**a**) annealed at 500 °C, (**b**) 600 °C and (**c**,**d**) 700 °C in N_2_ atmosphere.

SEM micrographs have been obtained for TFSOC in various annealing temperatures. Examination of either micrograph confirmed that increase the niobium content from 0.01 to 0.02 did not affect the surface morphology owing to its low content. Therefore, only the micrographs of TiSiNb_0.02_ TFSOC annealed at 500 °C (a), 600 °C (b) and 700 °C (c,d) are reported in Figure 4.

**Figure 5:**
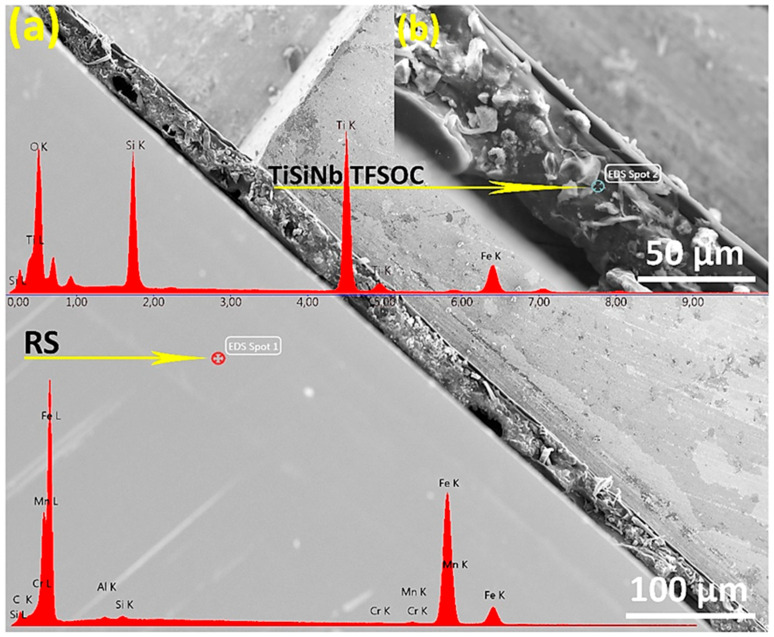
SEM micrograph (**a**) and an insert referred as (**b**) with EDS analysis of the polished cross-section of TiSiNb_0.02_ TFSOC annealed at 700 °C in N_2_ atmosphere.

Figure 4a reveals that the annealed TFSOC at 500 °C comprises continuous coating with just a few hairline micro-cracks, which demonstrated excellent thermal shock resistance at this temperature. However, as the annealing temperature increases from 600 °C (Figure 4b) to 700 °C (Figure 4c), the mesh of narrow elongated pits and cracks were observed in the TFSOC surfaces. As shown in Figure 4c, the second layer was also observed, from which might be concluded that different layers (multilayer) can be applied on the surface by the LbL technique to raise the thickness of the TFSOC composite [39]. The other reported that the annealing temperature can be effective on the pores and cavities of the coating surface as a significant factor [57]. The size of these network cracks (12–18 µm) is increased through raising the annealing temperature probably due to the release of –OH and water bonds to the surface (cf. TGA-DTA and FTIR analysis) [58]. These results do, however, demonstrate that the RS substrate is protected inhomogeneously by the massive flaky scale of TFSOC, which may have been manufactured due to surface tension between the TiSiNb gel and atmosphere through the drying and annealing process. The researchers have also shown that during the heat treatment process in the sol–gel technique the capillary forces produced are ultimately able to establish surface fractures [58,59,60]. Figure 4d demonstrates a TFSOC micrograph which specifically illustrates that the uniform coat of nanoparticles was constructed after annealing of 700 °C. In accordance with the FT-IR (cf. Figure 3) and XRD (further presented in Section 3.4) analyses, these observations might be attributed to titanium particles (anatase phase) mixed with iron oxide with an approximate size of 600 nm. Figure 5a. indicates the cross-sectional SEM micrograph of grinded RS doped with TFSOC annealed at 700 °C in an N_2_ atmosphere.

As shown in Figure 5b, the cross-sections approximately reveal 76 μm of TFSOC dense structure and microcrystals of titanium anatase phase (approx 10 μm) which is also evident on the surface (Figure 4d). In addition Figure 5a shows that the substrate is uniformly protected by TFSOC. There are also cavities between RS and TFSOC, which were randomly distributed in various sections. Since they did not reach on the surface, the area and length of these cavities are incalculable. It can be concluded that these microcavities were created during the LbL and drying steps by trapping decomposition products. This figure demonstrates also the EDS spectrum of cross-sectional RS (EDS spot 1) and TFSOC (EDS spot 2) annealed at 700 °C under N_2_ atmosphere. The EDS result of the RS was matched with Table 1. The regular and homogeneous surface morphology of TFSOC with Ti, Si and Fe compositions at the micro-level were observed. The results show the excellently-defined peak of 0.525 keV for OK element (At%: 29.96) that demonstrated the formation of solid oxide thin-film. TiK peaks at 4.5 keV and 4.75 keV were identified which indicate the titanium dioxide existence (At%: 33.05). The peaks at 0.2 keV and 1.75 keV were identified to confirm the presence of SiK (At%: 30.59). In addition, the peak was defined at 6.45 keV to confirm FeK (At%: 6.40), which indicated that the RS penetrates to the TFSOC owing to the high temperature of the annealing process. The TFSOC compositions obtained by EDS analysis corroborate that the overall Ti:Si ratios were identical to the ratios of the corresponding sols.

### 3.4. X-ray Diffraction Analyses

**Figure 6:**
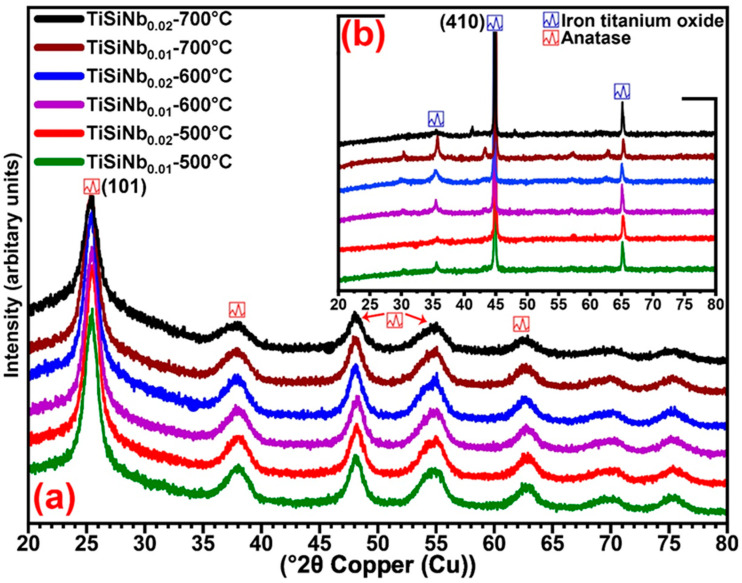
XRD patterns of TiSiNb powders (**a**) and TFSOC (**b**) at different annealing temperatures in N_2_ atmosphere.

The XRD patterns of TiSiNb powders (a) and TFSOC (b) annealed at various temperatures and Nb content in the N_2_ atmosphere are shown in Figure 6.

In compositions of TiSiNb powders (a), the results show that the increased temperature from 500 °C to 700 °C did not affect the peaks generated, which might be correlated with the formation and variations temperature in titanium and silicon phases [61]. It is evident that the increase in the annealing temperature resulted in increasing the intensity of the peaks and consequently forming crystallization. Only titanium oxide was detected and well-matched with the tetragonal anatase phase (01-071-1166) while silicon dioxide was not identified. In both Nb contents at the mentioned annealing temperature, the sharper peaks at 2θ = 25.34° were revealed to the (101) orientations. Considering the fact that the EDS analysis evidence demonstrates that coverings contain substantial quantities of silicon (At%: 30.59), however, due to the high annealing temperature, it can be concluded that the lack of silica composition in the XRD analysis might be attributed to its amorphous structure. To quantify the average size of TFSOC and TiSiNb nano-crystallite (L), the Scherrer Equation (4) [62] was utilized by K as a constant related to crystallite shape that is normally taken as 0.9 and XRD radiation of wavelength λ (nm) from measuring the full width at half maximum of peaks (β) in radian located at any 2θ in the pattern.

(4) $$L=\frac{K\times \lambda }{\beta \times \mathrm{Cos}ϴ}.$$

The average grain size of TiSiNb powders in different annealing temperatures (approx. 15 nm), was not changed with the increase of Nb content. Figure 6b demonstrates the XRD patterns of TFSOC at different annealing temperatures at two different Nb contents. The TFSOC XRD results were similar in all mentioned annealing temperatures. The results show that the Maghemite, titanium phase (Fe_2.18_O_4_Ti_0.42_, 01-084-1595), which demonstrates the iron oxide was formed on the surface. The combination of iron oxide and titanium phases was influenced by the pH of sol precursors in accordance with the pH-potential diagram of the iron–water system at RT [63] and annealing temperature. Low carbon steel such as C45E can progressively produce passive iron oxides in precursors with high pH [64]. Consequently, under the processes of annealing, these iron oxides were extracted and combined with TFSOC. Moreover, the XRD results of TFSOC shows that the transformation phases have not changed with increasing the annealing temperature and Nb content. The crystallite size of the maximum narrow peaks, which represented the high crystallinity of TFSOC was increased from 68 nm to 71 nm through increasing annealing temperature.

### 3.5. Studies on Corrosion

#### 3.5.1. Tafel Polarization Technique and Electrochemical Behavior

In all electrochemical techniques, I_corr_ can be used to determine the rate of corrosion from the measures of polarization. The Rp of annealed RS and TFSOC at different temperatures in SCS immersion after 1, 24, 360 and 720 h were determined using the Tafel extrapolation method from the intersection of cathodic and anodic Tafel curves. For this purpose, Rp was measured using the I_corr_, anodic (β_a_) and cathodic (β_a_) Tafel constants from the Stern-Geary Equation (5) as one of the most effective techniques.

(5) $$\mathrm{Rp}=\frac{\beta_{a}\times \beta_{c}}{2.303\times (\beta_{a}+\beta_{b})\times I_{\mathrm{corr}}}.$$

**Figure 7:**
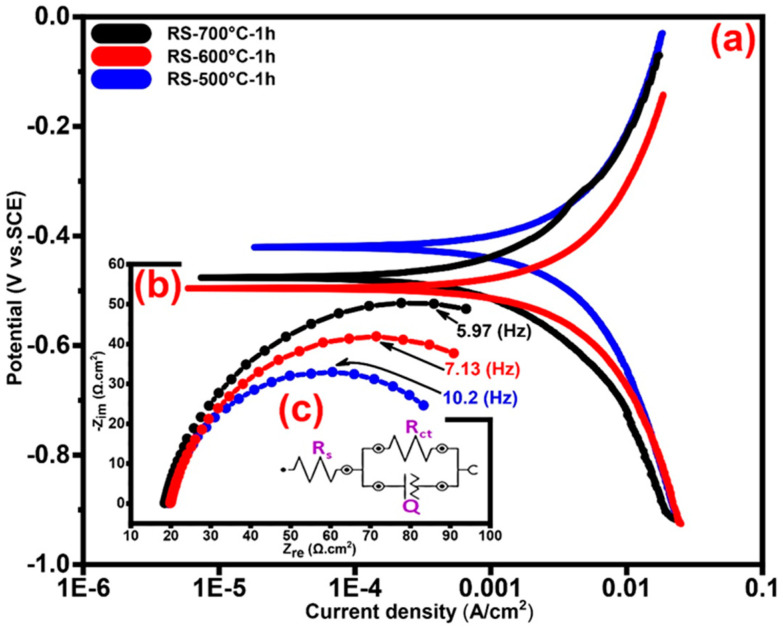
Potentiodynamic electrochemical polarization (PEP) (**a**), electrochemical impedance spectroscopy (EIS) (**b**) curves with equivalent electrical circuit (EEC) (**c**) of annealed RS at 500 °C, 600 °C and 700 °C in N_2_ atmosphere after 1 h immersion in standard condition solution (SCS).

In order to compare the corrosion behavior between the uncoated and coated RS, firstly the RS has been annealed at all mentioned temperatures in the N_2_ atmosphere. Then, the PEP and EIS tests of the annealed RS were performed in the SCS after 1, 24, 360 and 720 h immersion time. After 96 h of immersion, the results showed that the uncoated RS started to oxidatize and reduce the non-uniformity of the surface which can be assumed that the localized corrosion [65] occurred in the form of irregular and deep pitting. It can be concluded that this corrosion was primarily induced by the active–passive behaviour of iron due to the pH environment, regeneration the hydrogen ion, lack of protective black oxides such as Fe_3_O_4_ and chromium elements in the RS (cf. Figure 5 and Figure 6). Therefore, in PEP analyses under the assumption that the distribution of anode sites is uniform and its area is equivalent to the total sample surface area, the examination of the corrosion behaviour has not yielded accurate due to the RS surface oxidation and formation of the pitting corrosion [66]. Consequently, to compare the bare and coated RS, the PEP curves of the sample after 1 h immersion in the SCS were reported in Figure 7a and the results are shown in Table 3. 

**Figure 8:**
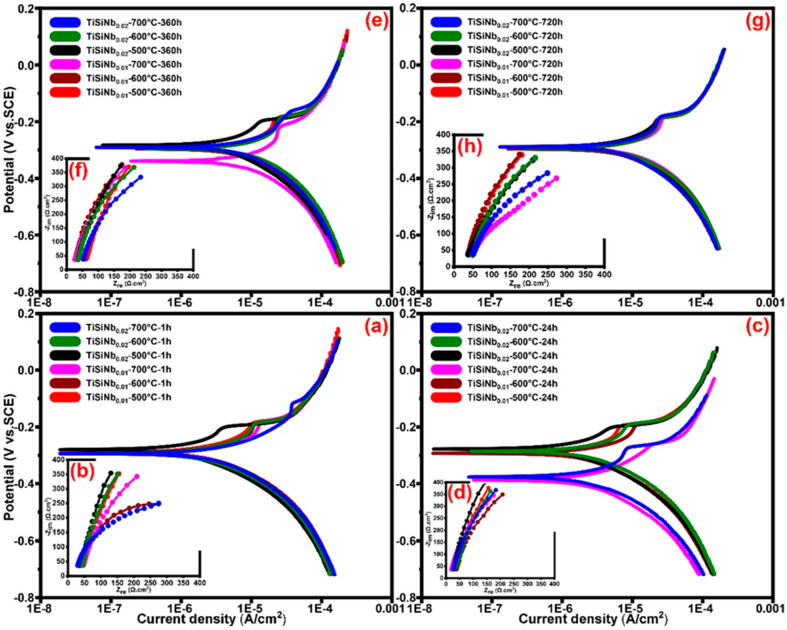
PEP (**a**,**c**,**e**,**g**), EIS (**b**,**d**,**f**,**h**) curves of annealed TFSOC at 500 °C, 600 °C and 700 °C in N_2_ atmospheres after immersion in SCS for 1, 24, 360 and 720 h.

The results indicate that the increased annealing temperature from 500 °C to 700 °C has improved the R_p_ from 326 Ω cm^2^ to 2030 Ω cm^2^, respectively. Therefore, it is conceivable to conclude that the increase in the annealing temperature resulted in the formation of the passive layer and thereby improved the RS corrosion resistance. Figure 8a,c,e,g shows the PEP curves of TFSOC with a different value of Nb annealed at 500 °C, 600 °C and 700 °C which were immersed in SCS for 1 (a), 24 (c), 360 (e) and 720 h (g), respectively. As the curves indicate, increasing the niobium dopant content is effective in improving the corrosion behavior of TFSOC due to its comprehensive performance such as excellent anti-corrosive performance. Therefore, based on the assumption that the results are almost the same only y_2_ = 0.02 is reported in Table 3.

Figure 8a shows the PEP curves of immersed TFSOC in SCS after 1 h. As can be seen, increasing the annealing temperature from 500 °C to 600 °C and 700 °C was decreased the E_corr_ from −28.1 V to −29.4 V and −29.5 V, respectively. Therefore, the Rp of TFSOC was decreased from 26,533 Ω cm^2^ to 13,588 Ω cm^2^ and 10,523 Ω cm^2^, respectively. The SEM micrograph reveals that TFSOC annealed at 500 °C (cf. Figure 4a) has fewer cracks compared to the rest of the samples. Therefore, there is the highest Rp of 26,533 Ω cm^2^ at this annealing temperature due to the non-cracking surface as one of the most important subjects to acts as a barrier and prevents corrosive ions from penetrating to the substrate. Figure 8b indicates the PEP curves of TFSOC after 24 h immersion in the SCS, which similarly decreased the Rp from 23,152 Ω cm^2^ to 9345 Ω cm^2^ by increasing the annealing mentioned temperature, respectively. As can be seen in Table 3 the similar results were obtained from 360 h (Figure 8e) and 720 h (Figure 8g) immersion in the SCS. Generally, the results demonstrated that the highest R_p_ of TFSOC was obtained from 500 °C annealed after being immersion in SCS for 1, 24, 360 and 720 h, respectively. Furthermore, as shown in Figure 8 and Table 3, after 720 h immersion, the maximum volume of decreased R_p_ was attributed to the TFSOC annealed at 500 °C. However, by comparing the other annealed temperatures, it can be concluded that the R_p_ has not decreased significantly after increasing the immersion time and their remains almost unchanged. As the annealing temperature increased, silicon dioxide is formed during the oxidation of silicon single crystal (cf. EDS result), as well as the titanium and iron oxide phases are grown. Then, due to the instability of silicon dioxide as a barrier the substrate oxides penetrated to the TFSOC surface through the nanoporous and cracks in the form of the combined titanium oxides (cf. Figure 6). Ultimately, to preserve the R_p_ stable, maybe such oxide phases behave similarly relying on the following mechanisms.

The long-term stability and superior corrosion resistance property of this TFSOC during the long-term immersion might be dependent to the variety of factors such as heat treatment atmosphere as well as the function of their electrochemical behaviour in the test environment. On the other hand, the researchers have realized that the process of heat treatment in the N_2_ atmosphere can increase the corrosion resistance of the bare substrate and coating [67,68]. Further, the reduction of dissolved nitrogen N_2_ according to Equation (6) can immediately be absorbed on the surface of TFSOC during the immersion in acidic environment consumes hydrogen ions and prevents acidification of the TFSOC surface by hydrolysis of metal ions and thereby prevents the destruction of TFSOC. As shown in EDS result (Figure 5a), there are various cavities between the RS and TFSOC which can be enriched by N_2_ through metal ion deposition during RS initial contact with the TiSiNb aged sols (dip-coating process) and film formation in the heat treatment process. Finally, the N_2_ is enriched in the metal at the substrate/TFSOC interface via cracks and nano-crevices, thus might be reducing the anodic dissolution rate and thus decreasing the I_corr_ (cf. Table 3), as a result, increasing the corrosion resistance. In the next mechanism, the passive films can be formed on the surface due to the consecutive immersion of the RS into the precursor sols and SCS or even during the PEP and EIS tests. In this case, both anode and cathode reactions are generally assumed as Equation (7) and Equation (8), respectively. However, rather than the metal and hydroxyl ions immediately combining to form a solid product shown in Equation (9), due to the SCS temperature and pH, these reactions (Equations (7) and (8)) constantly repeated, which resulted in the formation of the passive film. Therefore, firstly the metal ions react with hydroxyl ions and the intermediate complex has formed as shown in Equation (10). Then, water molecules surrounded these intermediate ions and the solid film has precipitated after the Equation (11) reaction. Resultantly, the water was produced as shown in Equation (12) owing to the combined the produced hydrogen ions in Equation (11) and remaining hydroxyl ions of cathode reaction from Equation (8). Finally, as shown in Equation (13) the solid metal hydroxide is converted to metal oxide due to the presence of water ions in the PEMFC operating environment.

(6) $$N_{2}+{4H}^{+}+3e\to {\mathrm{NH}}_{4}^{+}$$

(7) $$M\to M^{+2}+2e$$

(8) $${1/2O}_{2}+H_{2}O+2e\to {2\mathrm{OH}}^{-}$$

(9) $$M^{+2}+{2\mathrm{OH}}^{-}\to {M(\mathrm{OH}}_{2})$$

(10) $$M^{+2}+{\mathrm{OH}}^{-}\to {M(\mathrm{OH}}^{+})$$

(11) $$M\left({\mathrm{OH}}^{+}\right)+H_{2}O\to {M(\mathrm{OH})}_{2}+H^{+}$$

(12) $$H^{+}+{\mathrm{OH}}^{-}\to H_{2}O$$

(13) $${M(\mathrm{OH})}_{2}\to \mathrm{MO}+H_{2}O$$

Under this mechanism, freshly oxide formed film can contain a significant amount of water molecules, which can gradually decrease by continuous exposure to SCS. Eventually, the new oxide formed film progressively changes toward that of the metal with the loss of hydrogen ions. Following the formation of the metal oxide, the metal ions at a rate corresponding to the I_corr_ passed through the passive film. As a result, the corrosion resistance can be constant over a long time in the presence of corrosive ions in the SCS. Finally, it can be concluded that the TFSOC has demonstrated high corrosion and long-lasting resistance in the SCS, which is suitable for PEMFC_MI_ cost-effective applications.

#### 3.5.2. Electrochemical Impedance Spectroscopy (EIS)

The EIS technique was used to investigate the RS and TFSOC corrosion behavior after immersion in SCS. Investigating the characteristics of coatings protective on corrodible metals is one of EIS most usual applications. Figure 7b and Figure 8b,d,f,h show the Nyquist plots from EIS analyses of annealed RS (7b) and TFSOC after immersion in SCS for 1 (b), 24 (d), 360 (f) and 720 h (h) at a mentioned temperature in N_2_ atmosphere. As can be seen in Figure 7b, the low corrosion resistance of RS has not changed by increasing the annealing temperature. Therefore, because of the rapid oxidation of bare RS in the harsh environment, only the PEP results of 1 h at the mentioned annealing temperature are presented in this work. As shown in Figure 8b,d,f,h, the application of TFSOC have modified the shape and diameter of the semi-circles as compared to the RS plot (7b) which indicates an improvement of RS coated resistance. Furthermore, the Nyquist TFSOC plots demonstrate that the semicircular diameters were increased, which indicates a notable improvement of the corrosion resistance of the substrates after applying the TFSOC. The equivalent electrical circuit (EEC) has been used to obtain the parameter values of the RS and TFSOC Nyquist plots. As can be observed in Figure 1b, the gas and water flow channels are machined or stamped directly on the interconnect surface. Due to the presence of these channels, which are responsible for the homogeneous delivery and distribution of fuels, a significant portion of irreversible losses from ICR of approximately 11%, which clearly reduces PEMFC efficiency [69]. Therefore, to improve the efficiency and commercialization of PEMFCs, it is important to avoid the electrochemical degradation of the interconnectors. On other hand, good electrical conductivity ought to be the property of PEMFC_MI_. However, it seems that the high value of R_ct_ might have decreased the electroconductivity of PEMFC_MI_, which can have consequences for the efficiency of the interconnectors and eventually reduce the output power of the PEMFC. Therefore, to identify the TFSOC with good load transfer intensity in addition to reasonable corrosion resistance, the lowest values of the equivalent circuit parameter of EIS results, obtained from the curve-fitting method that has been used to model the bare and coated substrates, are compared. The optimum was found for the TiSiNb_0.02_ sample annealed at 700 °C. The data are reported in Table 4. The most appropriate circuit which has been matched precisely to the EIS results of RS and TFSOC with a low measurement error of impedance data (rel. std. error ≤0.14%) is schematically illustrated in Figure 7c. The Randles equivalent circuit [70] as one of the simplest and most common cell models can be used to extract information of EIS data from different protective thin-films. This model is often the basis for other more complex models which is commonly utilized when the Nyquist plot is in the form of a complete semicircle with no deviation from an ideal state. As shown in Figure 7c, the equivalent circuit involves the solution resistance (R_s_), charge transfer (R_ct_) and pseudocapacitance associated to the substrate-electrolyte interface (Q). In general, CPE is used to describe the capacitance, when the underlying electrochemical process is not described by a single relaxation frequency but by dispersion of relaxation frequencies (indicated by a flattened semicircle). It is specified by the (Y_0_) and the power index number (n) as provided in (Equation (14)); where Y_0_ is directly related to the active surface corrosion and electrical conductivity [71] and n is CPE power, which is attributed to the dispersion effects caused most probably by the roughness of the TFSOC surface [72]. Therefore, the identity and impedance of CPE are related to Equations (14) and (15), respectively.

(14) $$Y_{\mathrm{CPE}}=Y_{0}\times {\left(j\omega \right)}^{n}$$

(15) $$Z_{\mathrm{CPE}}=\frac{1}{Y_{0}\times {\left(j\omega \right)}^{n}}$$

where ω = 2πf (rad/s) as an angular frequency and j = −1^1/2^ represents the imaginary constant. Furthermore, the parameters Y_0_ or Admittance (Ω^−1^ cm^2^ s^n^) and n (−1 ≤ n ≥ 1) are independent of frequency. In general, the pure inductive, resistance and capacitive behaviors (RC) are attributed to the CPE with n value of −1, 0 and 1, respectively. As can be seen from Table 4, the TFSOC has the highest R_ct_ of 3.3 × 10^3^ Ω cm^2^ compared to the RS (179.15 Ω cm^2^) after 1 h immersion in the SCS. Over time, this value gradually decreased to the 2.7 × 10^3^ Ω cm^2^ (after 720 h), which caused a decrease of load transfer resistance.

**Figure 9:**
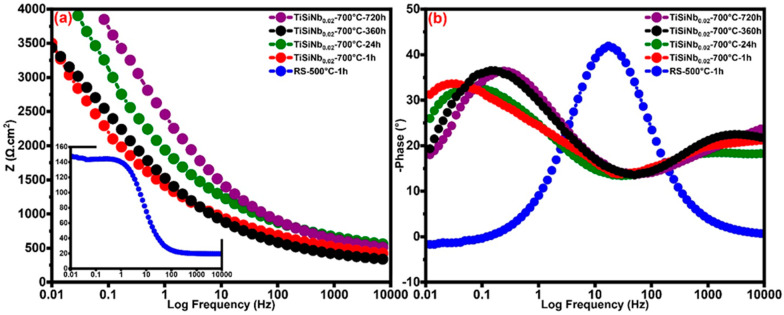
Bode plots (**a**) and Bode phase diagrams (**b**) for RS and TFSOC for different immersion time in SCS.

Formation of the high protective TFSOC with titanium oxide phase and its enhancement by iron oxide (cf. EDS and XRD) as well as the formation of the protective passive film (cf. PEP analyses) have resulted in the good constant resistance over the long immersion time. Decreasing the Y_0_ to 6.9 × 10^−3^ (Ω^−1^ cm^2^ s^n^) after 24 h immersion indicates that a small area on the TFSOC surface has participated in the corrosion process. Furthermore, according to the n values (between 0 and 1) as shown in Table 4, it can be concluded that the behavior of the CPE is a RC-type [38]. Hence, it can be assumed that owing to the thickening of the passive layers the R_ct_ value has not changed with increasing immersion time. Figure 9a,b show Bode plots and Bode phase diagrams of TFSOC at different immersion time in SCS, respectively. As shown in Bode plots of TFSOC three different regions as high (≥10^3^ Hz), medium (0.1–10^3^ Hz) and low (<10^3^ Hz) frequencies indicate the R_s_ properties, RC-type behavior and R_ct_ process at the SCS/electrode interface, respectively. As can be seen, despite long immersion time, the impedance parameters of the TFSOC were not significantly changed except that phase angle tended to decrease at low frequency. This slight decrease is due to the formation of pores/cracks in TFSOC surfaces (cf. Figure 4) which allows the occurrence of the localized corrosion in the SCS harsh environment. Nonetheless, as concluded by the EIS and PEP results, the SCS electrolyte cannot be used to diffuse and attack the substrate from these corrosion points. According to the passivation mechanism (cf. PEP studies), the passive layer of TFSOC generated by the corrosion products offered an extra protective barrier to the substrate, thereby hinder and retard electrolyte diffusion. It can be inferred that the TFSOC shows capacitive behavior with a significant phase angle between low/mid-frequency regions which can be correlated with thin-film insulation and compactness. Therefore, it can be concluded that the Nyquist plots from the EIS analyses were largely compatible with the PEP result and TFSOC can effectively protect the RS with little degradation of cathode working potential.

### 3.6. Computation and Evaluation of Conductivity and Activation Energy

**Figure 10:**
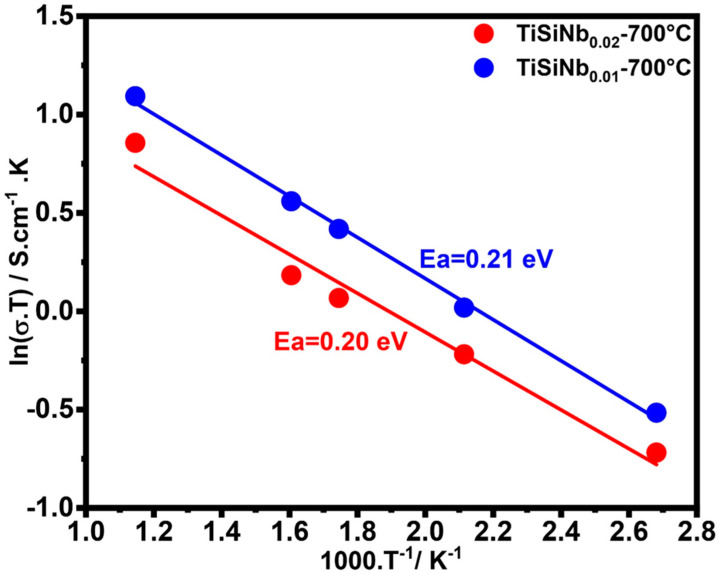
Arrhenius-plot of TiSNb with different Nb content annealed at 700 °C in N_2_ atmosphere.

Figure 10 illustrates the Arrhenius representation of Ti_0.50-y/2_Si_0.50-y/2_Nb_y1,2_O_2_ with two different Nb contents. It can be seen that for both samples the activation energy is around 0.20 eV. However, it appears that the concentration of Nb acts inversely on the TFSOC resistivity. If Nb as donor dopant would be compensated completely electronically, an increase of conductivity would be expected. From the decrease of conductivity with higher Nb content, it can be concluded that Nb at the higher content level is partially compensated by the formation of cation vacancies (V_Ti_′′′′). The activation energy of the conductivity gives evidence that the electrons generated by donor doping are trapped by either the formation of Ti^3+^ ions (in Kröger–Vink-notation Ti_Ti_′) or the association with oxygen vacancies. Since the number of oxygen vacancies in doped donor oxides should be very low, the first assumption seems more plausible. These trapped electrons are released either by hopping (polaronic motion) to neighbouring Ti^4+^ ions or by excitation to the conduction band. Unfortunately, the activation energy found in the literature for such Ti^3+^/Ti^4+^ hopping differs by one order of magnitude. Shan Yang et al. [73] estimated the activation energy for the intrinsic electron small polaron in rutile Ti oxide from electron paramagnetic resonance measurements as 0.024 eV, which would mean that these defects are thermally unstable at very low temperatures and would be fully ionized at ambient conditions. Di Valentin et al. [74], however, calculated the activation energy for the excitation of an electron from a six-fold-coordinated Ti_6c_^3+^ ion as 0.3 eV. One may take into account that the activation energy for polaron motion in a solid solution of Ti and Si oxides is increased because of the local distortion of the lattice due to the difference in the ionic radii and therefore is well above the suggested 0.024 eV of pure Ti oxide.

## 4. Conclusions

The TiSiNb TFSOC has been developed to improve the conductivity and electrochemical degradation resistance of C45E used as metal interconnects in PEMFC applications. Most parameters such as thermal stability, OH groups, phases and crystallite sizes can be controlled by the sol–gel method and annealing temperature. The SEM analysis shows the presence of a compact and homogeneous TFSOC mixed with iron oxide as evidenced by EDS and XRD analyses. The use of an N_2_ atmosphere and high annealing temperature leads to an increase in the self-protective and long-term stability of the TFSOC. To define their anti-corrosion efficiency, the highest long-term anti-corrosion activity after 720 h (1.33 kΩ cm^2^) immersion in SCS using the PEP has been obtained in comparison to RS. EIS data also further confirmed that the cheap C45E steel coated can play a significant function in improving the interconnect resistance. Due to the low activation energy, the material composition and heat treatment conditions could be optimized further to improve the conductivity. Owing to its homogeneity and high corrosion resistance in the PEMFC environment and cost-effective materials and method, the coated moderate C45E carbon steel can be considered as a cheap effective combination for interconnect in the future PEMFC technologies, which is effective for considerably increasing the PEMFC output power.

## Figures and Tables

**Table 1:** The C45E substrate chemical composition (wt.%).

Elements	Mn	C	Si	Cr	Ni	S	P	Fe
wt.%	0.65	0.45	0.27	0.25	0.25	0.02	0.04	balance

**Table 2:** Different annealing conditions and processing of the thin-film solid oxide coating (TFSOC) on the reference sample (RS) sample.

Annealing Temperature	Annealing Atmosphere	RS	Nb_0.01_	Nb_0.02_
700 °C	Nitrogen	RS-700 °C	TiSiNb_0.01_-700 °C	TiSiNb_0.02_-700 °C
600 °C		RS-600 °C	TiSiNb_0.01_-600 °C	TiSiNb_0.02_-600 °C
500 °C		RS-500 °C	TiSiNb_0.01_-500 °C	TiSiNb_0.02_-500 °C

**Table 3:** Electrochemical parameters of annealed RS and TFSOC in N_2_ atmospheres at mentioned temperatures after immersion for 1, 24, 360 and 720 h in SCS.

Sample	Immersion Time	Parameters				
		E_corr_ (V)	β_a_ (V/dec)	β_c_ (V/dec)	I_corr_ (A/cm^2^)	R_p_ (Ω cm^2^)
TiSiNb_0.02_-700 °C	720 h	−0.29.4	2.8 × 10^−2^	3.2 × 10^−2^	6.9 × 10^−7^	9262
TiSiNb_0.02_-600 °C		−0.29.5	3.3 × 10^−2^	3.3 × 10^−2^	7.1 × 10^−7^	9681
TiSiNb_0.02_-500 °C		−0.28.9	3.8 × 10^−2^	4.1 × 10^−2^	6.5 × 10^−7^	13,285
TiSiNb_0.02_-700 °C	360 h	−0.29.6	2.9 × 10^−2^	3.1 × 10^−2^	7.2 × 10^−7^	9038
TiSiNb_0.02_-600 °C		−0.29.6	4.1 × 10^−2^	4.2 × 10^−2^	9.5 × 10^−7^	9581
TiSiNb_0.02_-500 °C		−0.28.4	2.7 × 10^−2^	3.1 × 10^−2^	2.8 × 10^−7^	21,050
TiSiNb_0.02_-700 °C	24 h	−0.29.2	2.9 × 10^−2^	3.3 × 10^−2^	7.1 × 10^−7^	9345
TiSiNb_0.02_-600 °C		−0.28.7	3.9 × 10^−2^	4.1 × 10^−2^	7.1 × 10^−7^	12,423
TiSiNb_0.02_-500 °C		−0.27.9	3.6 × 10^−2^	4.2 × 10^−2^	3.7 × 10^−7^	23,152
TiSiNb_0.02_-700 °C	1 h	−0.29.5	3.1 × 10^−2^	3.9 × 10^−2^	7.1 × 10^−7^	10,523
TiSiNb_0.02_-600 °C		−0.29.4	3.6 × 10^−2^	4.1 × 10^−2^	6.1 × 10^−7^	13,588
TiSiNb_0.02_-500 °C		−0.28.1	2.9 × 10^−2^	3.5 × 10^−2^	2.6 × 10^−7^	26,533
RS-700 °C		−0.47.5	4.9 × 10^−2^	5.9 × 10^−2^	5.8 × 10^−6^	2030
RS-600 °C		−0.47.9	5.9 × 10^−2^	6.5 × 10^−2^	1.6 × 10^−5^	854
RS-500 °C		−0.48.4	5.6 × 10^−2^	3.7 × 10^−2^	3.1 × 10^−5^	326

**Table 4:** EIS fitting parameters of annealed RS at 500 °C and TFSOC at 700 °C after immersion in SCS.

Sample Name	RS-500 °C	TiSiNb0.02-700 °C			
*Immersion Time*	1 h	1 h	24 h	360 h	720 h
R_s_ (Ω cm^2^)	19.5	16.4	18.1	18.3	18.9
Y_0_ (Ω^−1^ cm^2^ s^n^)	2.9 × 10^−4^	2.4 × 10^−3^	6.9 × 10^−3^	6.5 × 10^−4^	1.5 × 10^−3^
n	0.9	0.6	0.6	0.8	0.7
R_ct_ (Ω cm^2^)	179.1	3.3 × 10^3^	2.8 × 10^3^	2.5 × 10^3^	2.7 × 10^3^

## References

[1] Rivarolo M., Rattazzi D., Lamberti T., Magistri L. (2020). Clean energy production by PEM fuel cells on tourist ships: A time-dependent analysis. Int. J. Hydrog. Energy.

[2] Alves-Lima D.F., Letizia R., Degl’Innocenti R., Dawson R., Lin H. (2020). Quantitative video-rate hydration imaging of Nafion proton exchange membranes with terahertz radiation. J. Power Sources.

[3] Hu M., Triulzi G., Sharifzadeh M. (2020). Technological change in fuel cell technologies. Design and Operation of Solid Oxide Fuel Cells.

[4] Park H., Kim D.-K., Kim H., Oh S., Jung W.S., Kim S.-K. (2020). Binder-coated electrodeposited PtNiCu catalysts for the oxygen reduction reaction in high-temperature polymer electrolyte membrane fuel cells. Appl. Surf. Sci..

[5] Jin J., He Z., Zhao X. (2020). Formation of a protective TiN layer by liquid phase plasma electrolytic nitridation on Ti–6Al–4V bipolar plates for PEMFC. Int. J. Hydrog. Energy.

[6] Shi J., Zhang P., Han Y., Wang H., Wang X., Yu Y., Sun J. (2020). Investigation on electrochemical behavior and surface conductivity of titanium carbide modified Ti bipolar plate of PEMFC. Int. J. Hydrog. Energy.

[7] Deyab M.A., Mele G. (2020). Stainless steel bipolar plate coated with polyaniline/Zn-Porphyrin composites coatings for proton exchange membrane fuel cell. Sci. Rep..

[8] Simaafrookhteh S., Khorshidian M., Momenifar M. (2020). Fabrication of multi-filler thermoset-based composite bipolar plates for PEMFCs applications: Molding defects and properties characterizations. Int. J. Hydrog. Energy.

[9] Kargar-Pishbijari H., Hosseinipour S.J., Aval H.J. (2020). A novel method for manufacturing microchannels of metallic bipolar plate fuel cell by the hot metal gas forming process. J. Manuf. Process.

[10] Bouziane K., Lachat R., Zamel N., Meyer Y., Candusso D. (2020). Impact of cyclic mechanical compression on the electrical contact resistance between the gas diffusion layer and the bipolar plate of a polymer electrolyte membrane fuel cell. Renew. Energy.

[11] Haye E., Deschamps F., Caldarella G., Piedboeuf M.-L., Lafort A., Cornil H., Colomer J.-F., Pireaux J.-J., Job N. (2020). Formable chromium nitride coatings for proton exchange membrane fuel cell stainless steel bipolar plates. Int. J. Hydrog. Energy.

[12] Pilinski N., Käding C., Dushina A., Hickmann T., Dyck A., Wagner P. (2020). Investigation of Corrosion Methods for Bipolar Plates for High Temperature Polymer Electrolyte Membrane Fuel Cell Application. Energies.

[13] Alo O.A., Otunniyi I.O., Pienaar H.C., Sadiku E.R. (2020). Electrical and mechanical properties of polypropylene/epoxy blend-graphite/carbon black composite for proton exchange membrane fuel cell bipolar plate. Mater. Today Proc..

[14] Song Y., Zhang C., Ling C.-Y., Han M., Yong R.-Y., Sun D., Chen J. (2019). Review on current research of materials, fabrication and application for bipolar plate in proton exchange membrane fuel cell. Int. J. Hydrog. Energy.

[15] Li X., Zhou P., Ogle K., Proch S., Paliwal M., Jansson A., Westlinder J. (2020). Transient stainless-steel dissolution and its consequences on ex-situ bipolar plate testing procedures. Int. J. Hydrog. Energy.

[16] Chen Z., Zhang G., Yang W., Xu B., Chen Y., Yin X., Liu Y. (2020). Superior conducting polypyrrole anti-corrosion coating containing functionalized carbon powders for 304 stainless steel bipolar plates in proton exchange membrane fuel cells. Chem. Eng. J..

[17] Leng Y., Ming P., Yang D., Zhang C. (2020). Stainless steel bipolar plates for proton exchange membrane fuel cells: Materials, flow channel design and forming processes. J. Power Sources.

[18] Li T., Zhang P.C., Liu K., Xu S., Han Y.T., Shi J.F., Sun J.C. (2019). Performance of Tantalum Modified 316L Stainless Steel Bipolar Plate for Proton Exchange Membrane Fuel Cell. Fuel Cells.

[19] Kumar N., Shaik G.P., Pandurangan S., Khalkho B., Neelakantan L., Chetty R. (2020). Corrosion characteristics and fuel cell performance of a cost-effective high Mn–Low Ni austenitic stainless steel as an alternative to SS 316L bipolar plate. Int. J. Energy Res..

[20] Chen G., Tao T., Gao P.-P., Xie Z.-Y., Wu X.-B. (2019). Corrosion Protection of Amorphous Carbon Coating for the Bipolar Plates of PEMFCs. Surf. Rev. Lett..

[21] Wilberforce T., Ijaodola O., Ogungbemi E., Khatib F.N., Leslie T., El-Hassan Z., Olabi A.G. (2019). Technical evaluation of proton exchange membrane (PEM) fuel cell performance—A review of the effects of bipolar plates coating. Renew. Sustain. Energy Rev..

[22] Jadi S.B., El Jaouhari A., Aouzal Z., El Guerraf A., Bouabdallaoui M., Wang R., Bazzaoui M. (2020). Electropolymerization and corrosion resistance of polypyrrole on nickel bipolar plate for PEM fuel cell application. Mater. Today Proc..

[23] Hickmann T., Zielinski O. (2020). Bipolar Plates: Different Materials and Processing Methods for Their Usage in Fuel Cells. E3S Web Conf..

[24] Liu S., Pan T.J., Wang R.F., Yue Y., Shen J. (2019). Anti-corrosion and conductivity of the electrodeposited graphene/polypyrrole composite coating for metallic bipolar plates. Prog. Org. Coat..

[25] Jiang L., Syed J.A., Lu H., Meng X. (2019). In-situ electrodeposition of conductive polypyrrole-graphene oxide composite coating for corrosion protection of 304SS bipolar plates. J Alloys Compd..

[26] Peng S., Xu J., Li Z., Jiang S., Munroe P., Xie Z.-H., Lu H. (2020). A reactive-sputter-deposited TiSiN nanocomposite coating for the protection of metallic bipolar plates in proton exchange membrane fuel cells. Ceram Int..

[27] Mahmoudi M., Raeissi K., Karimzadeh F., Golozar M.A. (2019). A study on corrosion behavior of graphene oxide coating produced on stainless steel by electrophoretic deposition. Surf. Coat. Technol..

[28] Lu J.L., Abbas N., Tang J., Hu R., Zhu G.M. (2019). Characterization of Ti_3_SiC_2_-coating on stainless steel bipolar plates in simulated proton exchange membrane fuel cell environments. Electrochem. Commun..

[29] Sadeghian Z., Hadidi M.R., Salehzadeh D., Nemati A. (2020). Hydrophobic octadecylamine-functionalized graphene/TiO_2_ hybrid coating for corrosion protection of copper bipolar plates in simulated proton exchange membrane fuel cell environment. Int. J. Hydrog. Energy.

[30] Hua Q., Zeng Y., He Z., Xu Q., Min Y. (2020). Microstructure, synergistic mechanism and corrosion behavior of tin oxide conversion film modified by chitosan on aluminum alloy surface. Colloid Interface Sci. Commun..

[31] Manso A.P., Marzo F.F., Garicano X., Alegre C., Lozano A., Barreras F. (2020). Corrosion behavior of tantalum coatings on AISI 316L stainless steel substrate for bipolar plates of PEM fuel cells. Int. J. Hydrog. Energy.

[32] Wang Y., Zhang S., Lu Z., Wang P., Ji X., Li W. (2018). Preparation and performance of electrically conductive Nb-doped TiO_2_/polyaniline bilayer coating for 316L stainless steel bipolar plates of proton-exchange membrane fuel cells. RSC Adv..

[33] Yang L., Qin Z.L., Pan H.T., Yun H., Min Y.L., Xu Q.J. (2017). Corrosion protection of 304 stainless steel bipolar plates of PEMFC by coating SnO_2_ film. Int. J. Electrochem. Sci..

[34] Park J.H., Jeon B.J., Lee J.K. (2015). Electrochemical characteristics of fluorine-doped tin oxide film coated on stainless steel bipolar plates. Surf. Coat. Technol..

[35] Huang K., Zhang D., Hu M., Hu Q. (2014). Cr_2_O_3_/C composite coatings on stainless steel 304 as bipolar plate for proton exchange membrane fuel cell. Energy.

[36] Mohammadi N., Yari M., Allahkaram S.R. (2013). Characterization of PbO_2_ coating electrodeposited onto stainless steel 316L substrate for using as PEMFC’s bipolar plates. Surf. Coat. Technol..

[37] Kamnerdkhag P., Free M.L., Shah A.A., Rodchanarowan A. (2017). The effects of duty cycles on pulsed current electrodeposition of ZnNiAl_2_O_3_ composite on steel substrate: Microstructures, hardness and corrosion resistance. Int. J. Hydrog. Energy.

[38] Khosravi S., Veerapandiyan V.K., Vallant R., Reichmann K. (2020). Effect of processing conditions on the structural properties and corrosion behavior of TiO_2_–SiO_2_ multilayer coatings derived via the sol–gel method. Ceram Int..

[39] Mumjitha M., Raj V. (2015). Fabrication of TiO_2_–SiO_2_ bioceramic coatings on Ti alloy and its synergetic effect on biocompatibility and corrosion resistance. J. Mech. Behav. Biomed. Mater..

[40] Wang Y., Zhang S., Wang P., Chen S., Lu Z., Li W. (2019). Electropolymerization and corrosion protection performance of the Nb:TiO_2_ nanofibers/polyaniline composite coating. J. Taiwan Inst. Chem. Eng..

[41] Wang Y., Zhang S., Lu Z., Wang L., Li W. (2018). Preparation and performances of electrically conductive Nb-doped TiO_2_ coatings for 316 stainless steel bipolar plates of proton-exchange membrane fuel cells. Corros Sci..

[42] Figueira R.B., Sousa R., Silva C.J.R. (2020). Multifunctional and smart organic–inorganic hybrid sol–gel coatings for corrosion protection applications. Adv. Smart Coatings Thin Film. Futur. Ind. Biomed. Eng. Appl..

[43] Wang D., Bierwagen G.P. (2009). Sol–gel coatings on metals for corrosion protection. Prog. Org. Coat..

[44] Nagode A., Jerina K., Jerman I., Vella D., Bizjak M., Kosec B., Karpe B., Zorc B. (2018). The effect of sol–gel boehmite coatings on the corrosion and decarburization of C45 steel. J. sol–gel Sci. Technol..

[45] Bai C.-Y., Wen T.-M., Hou K.-H., Ger M.-D. (2010). The bipolar plate of AISI 1045 steel with chromized coatings prepared by low-temperature pack cementation for proton exchange membrane fuel cell. J. Power Sources.

[46] Cui L.-Y., Cheng S.-C., Liang L.-X., Zhang J.-C., Li S.-Q., Wang Z.-L., Zeng R.-C. (2020). In vitro corrosion resistance of layer-by-layer assembled polyacrylic acid multilayers induced Ca–P coating on magnesium alloy AZ31. Bioact. Mater..

[47] Martin U., Ress J., Bosch J., Bastidas D.M. (2020). Stress corrosion cracking mechanism of AISI 316LN stainless steel rebars in chloride contaminated concrete pore solution using the slow strain rate technique. Electrochim. Acta.

[48] Abbas N., Shao G.N., Imran S.M., Haider M.S., Kim H.T. (2016). Inexpensive synthesis of a high-performance Fe_3_O_4_-SiO_2_-TiO_2_ photocatalyst: Magnetic recovery and reuse. Front. Chem. Sci. Eng..

[49] Biglu Y.F.G., Taheri-Nassaj E. (2013). Investigation of phase separation of nano-crystalline anatase from TiO_2_-SiO_2_ thin film. Ceram Int..

[50] Dihingia P.J., Rai S. (2012). Synthesis of TiO_2_ nanoparticles and spectroscopic upconversion luminescence of Nd^3+^-doped TiO_2_–SiO_2_ composite glass. J. Lumin..

[51] Kim Y.N., Shao G.N., Jeon S.J., Imran S.M., Sarawade P.B., Kim H.T. (2013). Sol–gel synthesis of sodium silicate and titanium oxychloride based TiO_2_–SiO_2_ aerogels and their photocatalytic property under UV irradiation. Chem. Eng. J..

[52] Zita J., Maixner J., Krýsa J. (2010). Multilayer TiO_2_/SiO_2_ thin sol–gel films: Effect of calcination temperature and Na^+^ diffusion. J. Photochem. Photobiol. A Chem..

[53] Yang Z., Xu Y., Yang S. (2016). Fabrication, characterization, and photocatalytic performance of TiO_2_ hybridized with SiO_2_. Russ. J. Appl. Chem..

[54] Yan S.-R., Gholami T., Amiri O., Salavati-Niasari M., Seifi S., Amiri M. (2020). Effect of adding TiO_2_, SiO_2_ and graphene on of electrochemical hydrogen storage performance and coulombic efficiency of CoAl_2_O_4_ spinel. J. Alloys Compd..

[55] Kapridaki C., Maravelaki-Kalaitzaki P. (2013). TiO_2_–SiO_2_–PDMS nano-composite hydrophobic coating with self-cleaning properties for marble protection. Prog. Org. Coat..

[56] Yang J., Liang Q. (2019). TiO_2_/SiO_2_ membrane materials via a sol–gel process: Preparation and characterization calcined under N_2_ atmosphere. Ferroelectrics.

[57] Koli V.B., Mavengere S., Kim J.-S. (2019). An efficient one-pot N doped TiO_2_-SiO_2_ synthesis and its application for photocatalytic concrete. Appl. Surf. Sci..

[58] Kirtay S. (2014). Characterization of SiO_2_-TiO_2_ Hybrid Corrosion Protective Coatings on Mild Steel. J. Mater. Eng. Perform..

[59] Krzak-Roś J., Filipiak J., Pezowicz C., Baszczuk A., Miller M., Kowalski M. (2009). The effect of substrate roughness on the surface structure of TiO_2_, SiO_2_, and doped thin films prepared by the sol–gel method. Acta Bioeng. Biomech..

[60] Halin D.S.C., Abdullah M., Mahmed N., Malek S.N.A.A., Vizureanu P., Azhari A.W. (2017). Synthesis and Characterization of TiO_2_/SiO_2_ Thin Film via sol–gel Method. IOP Conf. Ser. Mater. Sci. Eng..

[61] Riazian M., Montazeri N., Biazar E. (2011). Nano structural properties of TiO_2_-SiO_2_. Orient. J. Chem..

[62] Wiranwetchayan O., Promnopat S., Thongtem T., Chaipanich A., Thongtem S. (2020). Effect of polymeric precursors on the properties of TiO_2_ films prepared by sol–gel method. Mater. Chem. Phys..

[63] Gulyaev A., Kondrutieva O., Koldaev V., Koltsov V. Building a Three-dimensional Purbe Diagram. Proceedings of the 2020 IEEE Conference of Russian Young Researchers in Electrical and Electronic Engineering (EIConRus).

[64] Wongpanya P., Saramas Y., Chumkratoke C., Wannakomol A. (2020). Erosion–corrosion behaviors of 1045 and J55 steels in crude oil. J. Pet. Sci. Eng..

[65] Ziadi I., Alves M.M., Taryba M., El-Bassi L., Hassairi H., Bousselmi L., Akrout K. (2020). Microbiologically influenced corrosion mechanism of 304L stainless steel in treated urban wastewater and protective effect of silane-TiO_2_ coating. Bioelectrochemistry.

[66] Li Q., Dong L., Yang Y., Wu Z., Zhu H., Dong Y. (2020). Corrosion Behavior of AISI 1045 Carbon Steel in Metalworking Fluids Containing Pseudomonas xiamenensis. Int. J. Electrochem. Sci..

[67] Aversa A., Saboori A., Librera E., de Chirico M., Biamino S., Lombardi M., Fino P. (2020). The Role of Directed Energy Deposition Atmosphere Mode on the Microstructure and Mechanical Properties of 316L Samples. Addit. Manuf..

[68] Boes J., Röttger A., Becker L., Theisen W. (2019). Processing of gas-nitrided AISI 316L steel powder by laser powder bed fusion–Microstructure and properties. Addit. Manuf..

[69] Gu W., Baker D.R., Liu Y., Gasteiger H.A. (2010). Proton exchange membrane fuel cell (PEMFC) down-the-channel performance model. Handb. Fuel Cells.

[70] Ghorbani M.M., Taherian R., Bozorg M. (2019). Investigation on physical and electrochemical properties of TiN-coated Monel alloy used for bipolar plates of proton exchange membrane fuel cell. Mater. Chem. Phys..

[71] He R.Y., Jiang J., Wang R.F., Yue Y., Chen Y., Pan T.J. (2020). Anti-corrosion and conductivity of titanium diboride coating on metallic bipolar plates. Corros. Sci..

[72] Mani S.P., Rajendran N. (2017). Corrosion and interfacial contact resistance behavior of electrochemically nitrided 316L SS bipolar plates for proton exchange membrane fuel cells. Energy.

[73] Yang S., Brant A.T., Giles N.C., Halliburton L.E. (2013). Intrinsic small polarons in rutile TiO_2_. Phys. Rev. B.

[74] Di Valentin C., Pacchioni G., Selloni A. (2009). Reduced and n-type doped TiO_2_: Nature of Ti^3+^ species. J. Phys. Chem. C.

